# Cyclic Mechanical Strain Regulates Osteoblastic Differentiation of Mesenchymal Stem Cells on TiO_2_ Nanotubes Through GCN5 and Wnt/β-Catenin

**DOI:** 10.3389/fbioe.2021.735949

**Published:** 2021-11-15

**Authors:** Yanchang Liu, Wendan Cheng, Yao Zhao, Liang Gao, Yongyun Chang, Zhicheng Tong, Huiwu Li, Juehua Jing

**Affiliations:** ^1^ Department of Orthopaedics, The Second Hospital of Anhui Medical University, Hefei, China; ^2^ Sino Euro Orthopaedics Network, Berlin, Germany; ^3^ Shanghai Key Laboratory of Orthopaedic Implants, Department of Orthopaedics, Shanghai Ninth People’s Hospital, Shanghai Jiaotong University School of Medicine, Shanghai, China

**Keywords:** bone marrow stromal cells, TiO_2_ nanotube, osteogenic differentiation, GCN5 HAT, Wnt/β-catenin signaling pathway

## Abstract

Bone marrow mesenchymal stem cells (BMSCs) play a critical role in bone formation and are extremely sensitive to external mechanical stimuli. Mechanical signals can regulate the biological behavior of cells on the surface of titanium-related prostheses and inducing osteogenic differentiation of BMSCs, which provides the integration of host bone and prosthesis benefits. But the mechanism is still unclear. In this study, BMSCs planted on the surface of TiO_2_ nanotubes were subjected to cyclic mechanical stress, and the related mechanisms were explored. The results of alkaline phosphatase staining, real-time PCR, and Western blot showed that cyclic mechanical stress can regulate the expression level of osteogenic differentiation markers in BMSCs on the surface of TiO_2_ nanotubes through Wnt/β-catenin. As an important member of the histone acetyltransferase family, GCN5 exerted regulatory effects on receiving mechanical signals. The results of the ChIP assay indicated that GCN5 could activate the Wnt promoter region. Hence, we concluded that the osteogenic differentiation ability of BMSCs on the surface of TiO_2_ nanotubes was enhanced under the stimulation of cyclic mechanical stress, and GCN5 mediated this process through Wnt/β-catenin.

## Introduction

In the treatment of orthopedic diseases, the integration relationship between the implant prosthesis and the host bone has always been the main factor affecting the therapeutic effect and the life of the implant. Therefore, the integration of the implant prosthesis and the host bone on the interface and the promotion of host bone regeneration have become a momentous indicator for evaluating prostheses performance. The nature of the interaction between the joint prosthesis and host bone interface is actually the process of bone formation and resorption (Lepri et al., 2016). Bone mesenchymal stem cells (BMSCs) play an important role in bone formation. BMSCs are very sensitive to external mechanical stimuli. A recent study showed that the osteogenic differentiation ability of mesenchymal stem cells was enhanced on the surface of high-hardness titanium-related substrates (Tian et al., 2019).

Ti-based alloy prosthesis is one of the most commonly used joint prosthesis materials in orthopedics because of its high strength, low elasticity modulus, and good biocompatibility. Ti screws efficiently improved the level of Human bone marrow mesenchymal stem cells (hBMSC) mineralization at the bone–implant interface and induced osteogenic differentiation and increased osseointegration (Shi et al., 2017; Nichol et al., 2021). In this study, a dense oxide layer can be formed on the titanium surface through an electrochemical process. This oxide layer is composed of nanoscale tube-like structures under scanning electron microscopy. The key element is titanium dioxide (TiO_2_) (Necula et al., 2019). Our previous study demonstrated that TiO2 nanotubes can promote osteogenic differentiation of BMSCs (Tong et al., 2020). Many researches have shown that titanium metal can undergo extremely subtle deformation, and this prosthesis deformation caused by mechanical factors can affect the biological behavior of surface BMSCs, thus determining the differentiation direction (de Peppo et al., 2014; Uto et al., 2017; Leach et al., 2018). In the past, the research on the induction of BMSCs differentiation through mechanical signals mainly focused on the matrix material composition, the surface modification of matrix materials, and the stiffness of matrix materials, etc. (Jiang et al., 2018; Vainieri et al., 2020; Zhang et al., 2020). However, the substrate material itself still underwent slight elastic deformation after being implanted in the host. The regulation mechanism of this elastic deformation on the biological behavior of the cells on the surface is still unclear. Our previous studies have demonstrated that cyclic mechanical stress that caused specimen deformation can promote osteogenic differentiation of BMSCs on TiO_2_ nanotube-modified titanium substrates through self-developed cyclic mechanical stress load devices (Chang et al., 2019). By screening and testing the cyclic mechanical stress set by the gradient, the optimal cyclic mechanical stress parameter that promotes osteogenic differentiation is determined. However, the mechanism of the signal pathway that mechanical cyclic stress promotes the osteogenic differentiation of BMSCs is still not clear, and further exploration is needed.

In the osteogenic differentiation of BMSCs, the Wnt/β-catenin signaling pathway is one of the most important pathways. The classical Wnt/β-catenin pathway stimulates the expression of alkaline phosphatase (ALP), osteopontin (OPN), osteocalcin (OCN), and runt-related transcription factor 2 (RUNX2) to interfere with the whole process (Krause et al., 2010). Previous researches claimed that mechanical signaling can influence the osteogenic differentiation of BMSCs through regulating the canonical Wnt pathway. Appropriate mechanical signal stimulation can increase the expression of Wnt gene, facilitate osteogenic differentiation, and reduce bone loss (Song et al., 2017).

The activation and inhibition of signaling pathways can be affected by histone acetylation modification (Wu et al., 2017). In the nucleus, histone acetylation and deacetylation are in a dynamic equilibrium. The process is regulated by histone acetyltransferases (HATs) and histone deacetylases (HDACs). Most of the current research on histone acetylation is concentrated on HDACs (Emori et al., 2012; Wang et al., 2015). HDACs remove the acetyl group from histones and combine histones with negatively charged DNA tightly. This modification makes chromatin to be more compact such that gene transcription could be inhibited. While HAT has the opposite function which could transfer the acetyl group of acetyl-CoA to the specific lysine residue at the amino terminus of histones, it would make the chromatin sparse and promote gene transcription (Han et al., 2016). HDAC is mechanosensitive and could inhibit osteogenic differentiation and decrease the level of BMSC mineralization through the Wnt signaling pathway. Regarding the role of HDACs in bone differentiation, many studies using HDAC inhibitors or RNA interference have identified a series of enzymes acting as regulators of bone formation (Desiderio et al., 2014; Sato et al., 2020). However, there are few reports on the regulation of the HATs family. As one of the important members of the HATs family, GCN5 has been confirmed to promote the osteogenic differentiation of BMSCs through multiple signaling pathways (Wang et al., 2018; Zhao et al., 2018; Sakai et al., 2016). However, whether GCN5 can be regulated by mechanical signals to affect the activity of Wnt/β-catenin signaling pathway and osteogenic differentiation of BMSCs has not been reported.

In this study, we used rat bone marrow mesenchymal stem cells as the research object to observe the effects of cyclic mechanical stress on the expression of GCN5 and the activity of classical Wnt/β-catenin signaling pathway and osteogenesis differentiation of BMSCs on the surface of TiO_2_ nanotube–modified titanium matrix. On the basis of previous research, we will explore the mechanism on the epigenetic level in-depth and try to provide new insights into the regulation of cyclic mechanical stress on the osteogenic differentiation of rat BMSCs.

## Materials and Methods

### Isolation and Culture of BMSCs

Sprague Dawley (SD) rats (4 weeks old) were purchased from the Experimental Animal Center of Anhui Medical University (SPF grade, weighed 100–130 g) (Hefei, China). A total of ∼50 rats were used for various experimental approaches in the current study. The rats were housed in groups (four to five rats per cage). All rats received a standard rodent diet and tap water ad lib. A constant temperature of 21–23°C and humidity of 50 ± 5% were maintained. Following feeding for 1 week, the rats were killed by cervical dislocation and were confirmed to have no spontaneous breathing and heartbeat. All efforts were made to minimize suffering. The rats were immersed in 75% ethanol and sterilized for 20 min. After removing the femur under aseptic conditions, scissors were used to cut both ends of the femur. A 5-ml syringe equipped with a 25-gauge needle was used to rinse the bone marrow in the medullary cavity with α-modified minimum essential medium (α-MEM, HyClone, Invitrogen, United States). The washed cells were collected, cultured in α-MEM supplemented with 10% fetal bovine serum (Gibco, United States) and 1% penicillin/streptomycin (Gibco, United States), and incubated at 37°C with 5% CO_2_. All experiments used cells from passages 3 to 5. The study has been approved by the Ethics Committee of the Second Affiliated Hospital of Anhui Medical University.

### Specimen Preparation of TiO_2_ Nanotubes

A pure titanium foil with a thickness of 2 mm was cut into a square with a length of 35 mm and used as a substrate. The top and bottom surfaces of the foil were sanded with silicon carbide sandpaper. A pure titanium foil was soaked in absolute ethanol and cleaned with an ultrasonic cleaner. After washing again with deionized water, the foil was air-dried at room temperature. The nanotubes were formed in electrolyte solution containing 0.15 M NH_4_F at 20 V for 30 min by anodization using a potentiostat. The cathode was a pure platinum foil. After anodization was completed, the sample was rinsed with deionized water.

### Cyclic Mechanical Stress Loading Application

**FIGURE 1:**
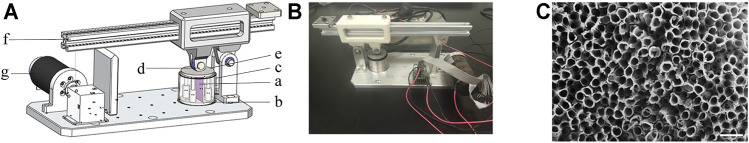
Cyclic mechanical loading device and TiO_2_ nanotubes topography. **(A)** The diagrams of the mechanical loading device. **(B)** Picture of the real product. **(C)** Surface titanium dioxide nanotubes are approximately 80 nm in diameter. Scale bars: 200 nm.

BMSCs were seeded on the TiO_2_ nanotubes–modified titanium specimens at a density of 1 × 104 cells/cm^2^. The cells were cultured for 48 h to allow them to attach and reach approximately 80% confluence, after which the growth medium was replaced, and cyclic mechanical stress was applied. The cyclic mechanical stress parameters used for our manufactured mechanical loading device (Figures 1A,B) are as follows: 0.3 and 0.9% elastic strain magnitude, 5-Hz sinusoidal curve, for 30 min/day (Chang et al., 2019). The specimen (a) with BMSCs cultured on the surface was placed into the fixed slot (b) and fixed. Then, the fixing slot containing the specimen was put into the fixed cylinder (c). The medium was added to the fixed cylinder until the test piece became submerged, so as to ensure that the specimen and the cells on the surface were always immersed in the medium during the process of applying cyclic mechanical stress. The top hammer (d) applied cyclic mechanical stress by pressing the top cover (e) on the top of the fixed slot. The top hammer was connected with the winch (g) through the lever (f). The device was connected through the computer, the winch rotation parameters were set, and the device was started. When the specimen is compressed, the elastic strain magnitude is ((the original height-the height after compression)/the original height) (0.3% and 0.9%). The entire process took 7 days. Cells were harvested immediately after the mechanical stress stimulation was applied.

### DKK1 Treatment

To study osteogenic differentiation with DKK1, the density of 1 × 104 cells/cm^2^ was seeded to a specimen. The conditioned medium was changed with DKK1 (100 ng/ml, 120–30, Pepro-Tech, United States). The medium was changed every 3 days. The cells were harvested after 7 days of culture for other experiments.

### Lentiviral Vector Construction and Transduction

We used PCR to amplify Rat GCN5. XbaI and NotI were used to digest the amplicons before inserted into the pCDH lentiviral vector. The sequences of the primers were forward: AGC​ACT​CCC​ATC​TTC​AGT​CC and reverse: GCT​TCC​TCT​TCT​CTC​CTG​GCA​T. The primers for GCN5 shRNA were forward: CCG​GGC​AGG​GTG​TTC​TGA​ACT​TTC​TCA​AGA​GAA​AAG​TTC​AGA​ACA​CCC​TGC​TTT​TTT​G and reverse: ATT​CAA​AAA​AGC​AGG​GTG​TTC​TGA​ACT​TTT​CTC​TTG​AGA​AAG​TTC​AGA​ACA​CCC​TGC.

Restriction enzymes AgeI and EcoRI were used to digest the PCR product before incorporated into the pLKD vector. Sanger sequencing was performed to verify the inserted fragments. To produce the lentivirus, 293T cell line was co-transfected with two packaging vectors (psPAX2 and pMD2.G) and two plasmid vectors. The supernatant was centrifuged at 1,000 rpm for 10 min to get rid of the cell debris. A 0.45-μm polyethersulfone low-protein-binding filter was used to filter. The titer of the lentivirus was 115 IFU/ml, assessed by a quantification kit (Invitrogen, United States). A vector inserted with scrambled GCN5 was used as a negative control.

### ALP Staining

BMSCs were seeded onto TiO_2_ nanotubes–modified titanium specimens, and cyclic mechanical stress was applied. On day 7, ALP activity was measured using an ALP kit (Hongqiao, China) according to the manufacturer’s instructions. The results were observed under a stereo microscope.

### Real-Time PCR

Total RNA was extracted from the cells with TRIzol (Invitrogen, United States) and reverse-transcribed into cDNA using a PrimeScriptTM cDNA Synthesis kit (6210A, Takara, Japan) according to the manufacturer’s instructions. Real-time PCR was performed with SYBR^®^ Premix Ex Taq™ (Takara, Japan). GAPDH served as an internal control, and the expression level relative to GAPDH was calculated using 2−ΔΔCq method. The primer sequences are listed in Table 1.

**TABLE 1:** Sense and anti-sense primers for real-time PCR.

Genes	Forward(5′-3′)	Reverse(5′-3′)
GCN5	AGC​ACT​CCC​ATC​TTC​AGT​CC	CTT​CCT​CTT​CTC​TCC​TGG​CAT
Axin-2	TCC​TTA​CCG​CAT​GGG​GAG​TA	GTG​GGT​TCT​CGG​GAA​GTG​AG
Wnt1	CGA​TGG​TGG​GGC​ATC​GTG​AA	TGC​CAC​TTG​CAC​TCT​CGA​AC
Wnt6	GCA​GCA​GGA​CAT​TCG​AGA​GA	TGA​CAA​CCA​CAC​TGT​AGG​AGC
Wnt10a	CCC​CAT​CTT​CAG​CAG​AGG​TTT	CGC​AAG​CCT​TCA​GTT​TAC​CC
Ocn	TCA​ACA​ATG​GAC​TTG​GAG​CCC	GCA​ACA​CAT​GCC​CTA​AAC​GG
Opn	CCA​GCC​AAG​GAC​CAA​CTA​CA	AGT​GTT​TGC​TGT​AAT​GCG​CC
Runx2	GCG​GTG​CAA​ACT​TTC​TCC​AG	CCT​TAA​ATA​TTA​CTG​CAT​GGA​CTG​T
GAPDH	TTTAGGGGCGCTGGTGC	TAC​GGC​CAA​ATC​CGT​TCA​CA

### Western Blot

The cells were washed three times with PBS and lysed with RIPA buffer supplemented with protease and phosphatase inhibitors for 30 min at 4 C. For the Western blot analysis, 15 μL of the sample was separated by 12% sodium dodecyl sulfate–polyacrylamide gel electrophoresis and electro-transferred onto nitrocellulose membranes. The primary antibodies used were rabbit polyclonal anti-GCN5 (1:1000, ab231075, Abcam, China); rabbit monoclonal anti-Axin-2 (1:1000, ab109307, Abcam, China); rabbit monoclonal anti-β-catenin (1:1000, ab32572, Abcam, China) and mouse monoclonal phosphorylated β-catenin (1:500, 05-665-AF647, Millipore, United States); rabbit monoclonal anti-H3K9ac (1:1000, ab10812, Abcam, China); rabbit monoclonal anti-RUNX2 (1:1000, ab192256, Abcam, China); rabbit monoclonal anti-OCN (1:1000, ab102936, Abcam, China); and rabbit monoclonal anti-OPN (1:1000, ab63856, Abcam, China). For normalization of protein loading, GAPDH antibody (1:1000, ab245357, Abcam, China) was used. The protein bands were measured with ImageJ 1.48v software and normalized to the corresponding GAPDH bands. The relative density of each target protein normalized to the control was used to represent the changes in expression of target proteins.

### ChIP Assay

ChIP assay was performed following the manufacturer’s instructions of a ChIP assay kit (Millipore, United States). The rabbit monoclonal anti-GCN5 (1:1000, ab231075, Abcam, China) and anti-H3K9ac (1:1000, ab10812, Abcam, China) were used. The rat IgG (I8015, Millipore, United States) and anti-RNA polymerase II (PLA0127, Millipore, United States) were used as the negative and positive control, respectively. The precipitated DNA samples were assessed by real-time PCR. Primers were designed from the region of 500 bp distance to the transcription start sites. The primer sequences are listed in Table 2. The values of the results were normalized to the input value.

**TABLE 2:** Sense and antisense primers for ChIP real-time PCR.

Genes	Forward(5′-3′)	Reverse(5′-3′)
Wnt1	AAC​TCC​ACC​CAT​GCT​CTG​T	GCT​GTG​GTC​CCT​TCT​CTT​CC
Wnt6	CAG​GGA​CCC​GTA​GAC​AAG​TG	TAT​TGG​GGG​CGG​ACA​GTG​TA
Wnt10a	CAT​TCA​GGT​TAG​GGC​CCC​AG	CAC​ATT​TGT​CTT​TGG​GCT​TCA​TCT

### Statistical Analysis

GraphPad Prism 7.0 (GraphPad Software) was used for statistical analysis. Data are expressed as mean ± SEM. Student's t test was used for comparing two groups, one-way analysis of variance was used for comparing multiple groups, and the least-significant difference method was used as a post-hoc test for multiple comparisons between groups. *p* < 0.05 was considered as significant.

## Results

### Effect of Cyclic Mechanical Strain on Osteogenic Differentiation of BMSCs

**FIGURE 2:**
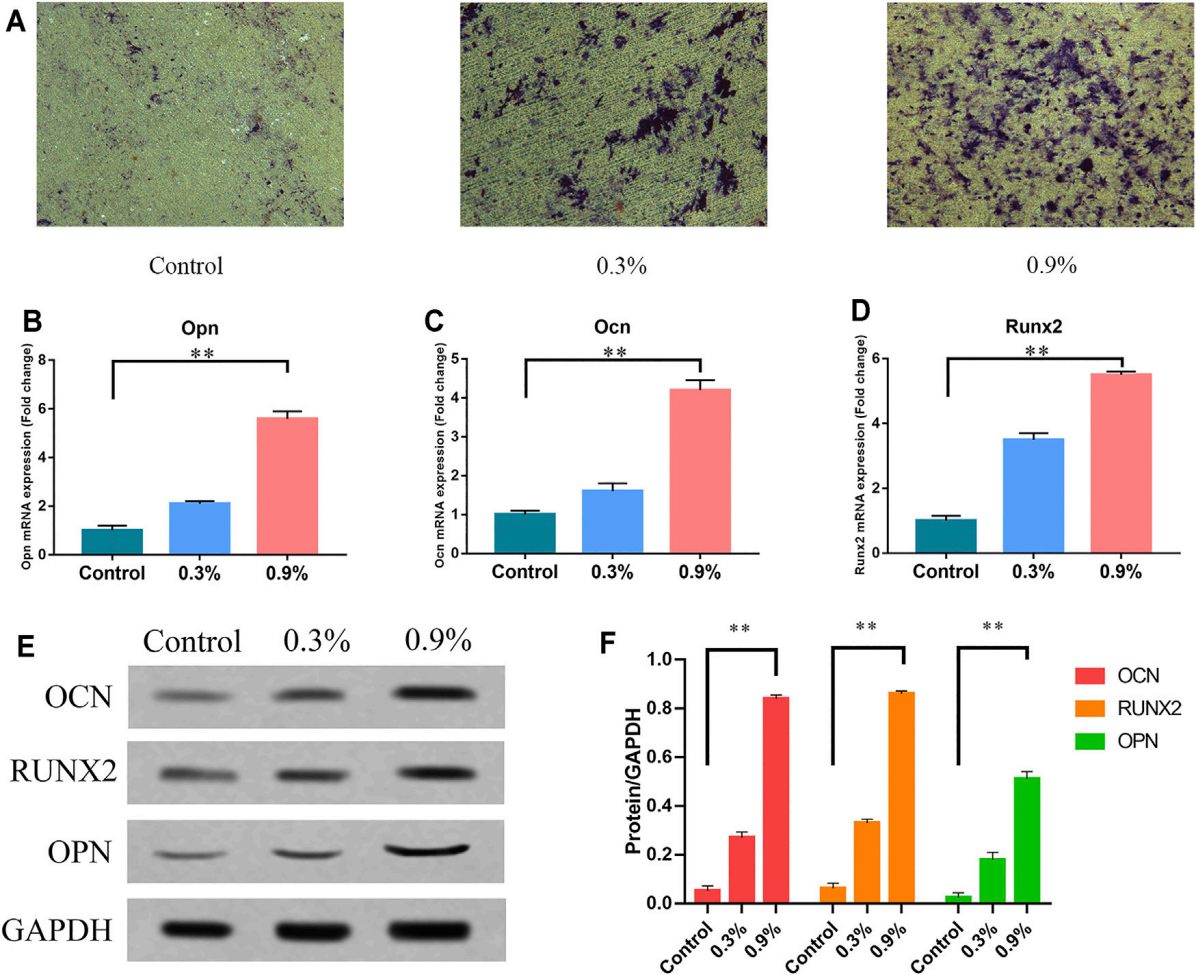
Cyclic mechanical strain can promote osteogenic differentiation of BMSCs. **(A)** ALP activity of BMSCs after 7 days of treatment. Scale bars: 500 μm. **(B–D)** The mRNA expression of Opn, Ocn, and Runx2 in BMSCs after 7 days of treatment. RT-qPCR data are presented as mean ± SD normalized against the control group data. The housekeeping gene GAPDH was used as an internal control. **(E)** Western blots of Opn, Ocn, and Runx2 after 7 days of treatment. GAPDH is shown as the loading control. **(F)** Semi-quantitative and statistical analysis of the results in Panel E. Values are plotted as mean ± SD, *p < 0.05, **p < 0.01.

Our previous studies have measured the topography of the TiO_2_ nanotube–modified titanium specimen surface and related physical parameters (Chang et al., 2019). The topography of the specimen surface is shown in Figure 1C. The schematic diagram and model diagram of the cyclic mechanical strain load device are showed in Figures 1A,B. The diameter of TiO_2_ nanotubes is about 80 nm (Figure 1C). We applied three levels of cyclic mechanical elastic strain to titanium sheets planted with BMSCs. After 7 days of continuous treatment, we found that significantly higher levels of ALP activities in the 0.3 and 0.9% groups, especially in the 0.9% group (Figure 2A). Then the results of real-time PCR indicated that the mechanical strain promoted the expression of osteogenesis genes, such as Runx2, OPN, and OCN (Figures 2B–D). The results of the Western blot showed that mechanical strain increased the expression of these osteogenic markers from the protein level (Figure 2E). These results further validate our previous research findings (Chang et al., 2019). These indicated that rat BMSCs are sensitive to external mechanical signal stimulation, and the elastic deformation caused by cyclic mechanical strain on titanium specimen can promote the osteogenic differentiation of BMSCs.

### Regulation of Cyclic Mechanical Stress on the Activity of Wnt/β-Catenin Signaling Pathway in BMSCs

Previous literature reports that high levels of Wnt signaling, an important signaling pathway that regulates osteogenic differentiation, can be activated in osteogenic differentiation of BMSCs and promote bone formation (Wang et al., 2017; Zhu et al., 2019). In osteoporotic mice, the level of Wnt ligand protein was reduced, and the activity of the entire pathway was decreased (Karadeniz et al., 2020). Therefore, the Wnt/β-catenin signaling pathway is a critical signaling pathway in osteogenic differentiation of BMSCs. Wnt acted as an activator of the entire signaling pathway and bound to the membrane surface ligand complex to activate the pathway.

**FIGURE 3:**
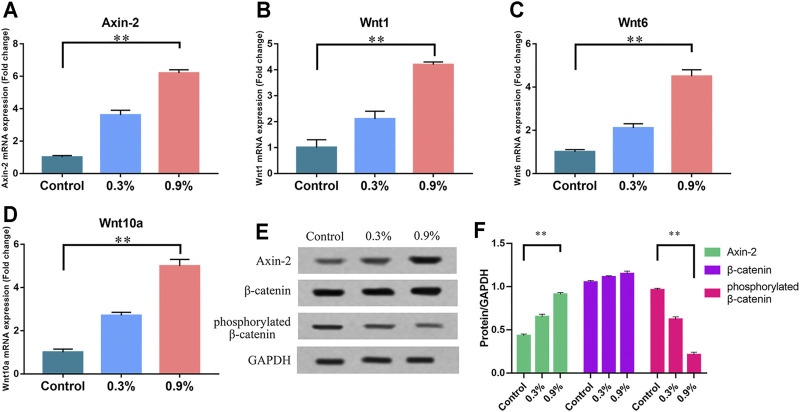
Mechanical strain can increase the activity of the Wnt/β-catenin signaling pathway in BMSCs. **(A–D)** The mRNA expression of Axin-2, Wnt1, Wnt6, and Wnt10a in BMSCs after 7 days of treatment. RT-qPCR data are presented as mean ± SD normalized against the control group data. The housekeeping gene GAPDH was used as an internal control. **(E)** Western blots of Axin-2, phosphorylated β-catenin, and β-catenin after 7 days of treatment. GAPDH is shown as the loading control. **(F)** Semi-quantitative and statistical analysis of the results in Panel E. Values are plotted as mean ± SD, *p < 0.05, **p < 0.01.

Wnt signaling pathway activity was observed under mechanical strain and nonmechanical strain. Western blot and real-time PCR were used to detect key molecules in the Wnt/β-catenin signaling pathway: Wnt1, Wnt6, Wnt10a, β-catenin, phosphorylated β-catenin [phosphorylated β-catenin could be regarded as inactive because it was about to be degraded (Zhu et al., 2019)], and Axin-2 [a defined target in the Wnt/β-catenin signaling pathway (Zhu et al., 2019)]. Western blot revealed that the level of phosphorylated β-catenin was lower and Axin-2 was higher in the 0.9% group after 7 days of exposure to mechanical strain. The level of β-catenin was stable simultaneously (Figures 3E,F). Real-time PCR indicated that the transcription of Wnt1, Wnt6, Wnt10a, and Axin-2 in the 0.9% group was significantly increased compared with that of the control group (Figures 3A–D). These results indicate that the Wnt/β-catenin signaling pathway was activated by mechanical strain and played a key role in the osteogenic differentiation of BMSCs.

### Influence of Cyclic Mechanical Stress on Histone Acetyltransferase GCN5

**FIGURE 4:**
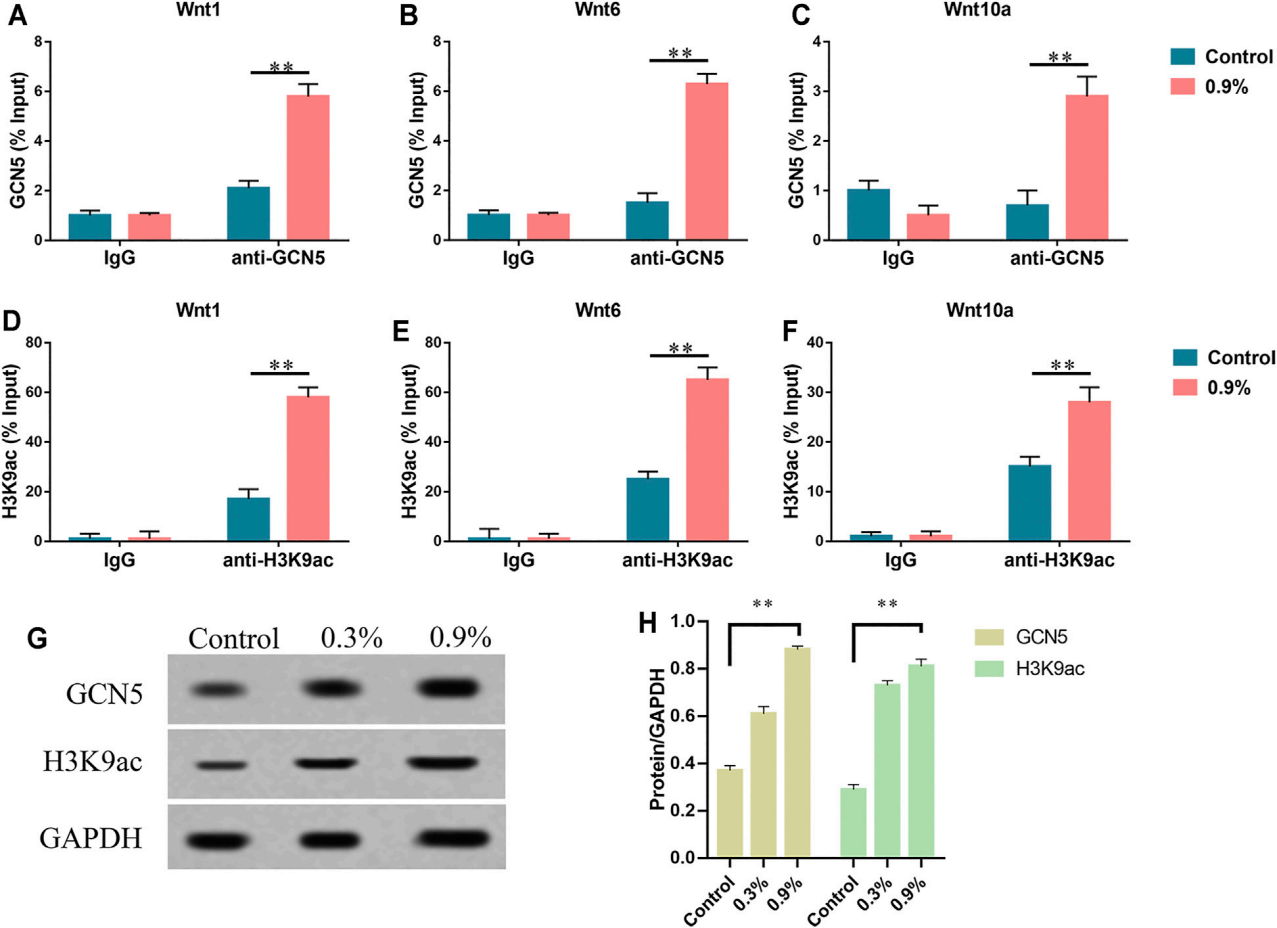
Histone acetyltransferase GCN5 is increased due to mechanical strain. **(A–C)** The ChIP assay was performed to evaluate the combination of GCN5 and IgG to Wnt1, Wnt6, and Wnt10a promoters in BMSCs which were exposed to mechanical strain. **(D–F)** The ChIP assay was performed to evaluate the enrichment of H3K9ac and IgG to Wnt1, Wnt6, and Wnt10a promoter regions. **(G)** Western blots of GCN5 and H3K9ac after 7 days of treatment. GAPDH is shown as the loading control. **(H)** Semi-quantitative and statistical analyses of the results in Panel G. Values are plotted as mean ± SD, *p < 0.05, **p < 0.01.

After applying mechanical strain to the titanium specimen, we detected the expression level of GCN5 in BMSCs through real-time PCR and Western blot. GCN5, one of the important members of the HATs family, played a significant regulatory role in a variety of signaling pathways, especially the Wnt signaling pathway (Li et al., 2016; Karadeniz et al., 2020). As the elastic deformation aggravated, the expression level of GCN5 gradually increased. Correspondingly, H3K9 was the major target site of GCN5 (Karadeniz et al., 2020). H3K9 became H3K9ac after being acetylated. The results showed that H3K9ac had the same trend as GCN5 and elastic strain promoted H3K9 acetylation (Figure 4G). In addition, we performed ChIP-qPCR to demonstrate the direct effects of GCN5 and H3K9ac with the Wnt gene expression. The ChIP analysis showed that the enrichment of GCN5 and H3K9ac in the Wnt1, Wnt6, and Wnt10a promoter regions was increased in the 0.9% group (Figures 4A–F, Supplementary Figure S1 and Supplementary Table S1). These results indicate that mechanical strain can promote the expression of GCN5 and increase the level of acetylation of H3K9 by promoting GCN5 binding to the promoter region of Wnt gene.

### The Effect of GCN5 Regulated by Cyclic Mechanical Stress on Osteogenic Differentiation of BMSCs

**FIGURE 5:**
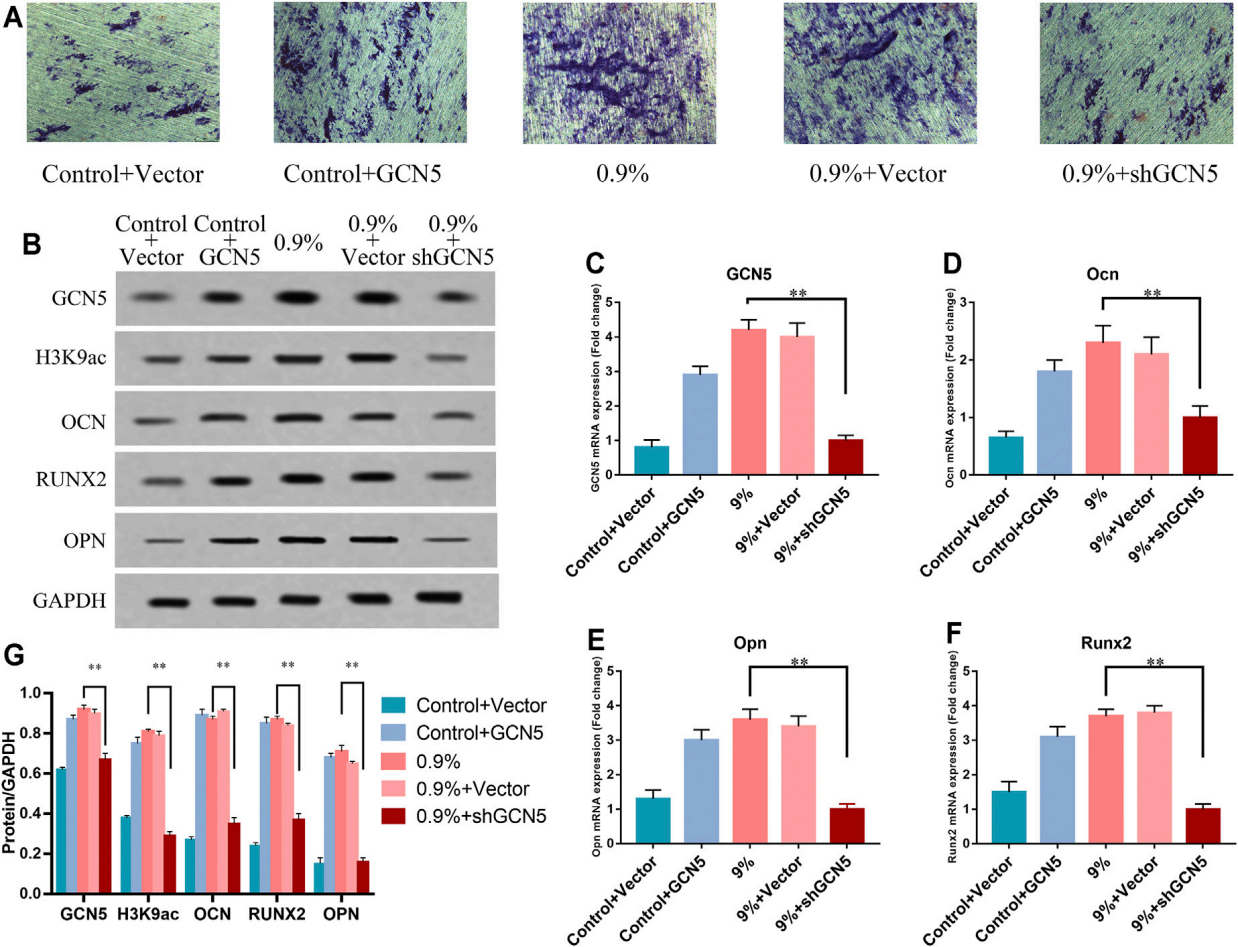
The increased level of GCN5 caused by mechanical strain promoted osteogenic differentiation of BMSCs. **(A)** ALP activity of BMSCs transfected with overexpression of GCN5 in the control group or shRNA of GCN5 in the 0.9% group. Scale bars: 500 μm. **(B)** Western blots of GCN5, H3K9ac, Ocn, Opn, and Runx2 in BMSCs transfected with the overexpression of GCN5 in the control group or shRNA of GCN5 in the 0.9% group. GAPDH is shown as the loading control. **(C)** The mRNA expression of GCN5 verified gain- and loss-of-function assays promoted by suppressing the expression of GCN5. **(D–F)** The mRNA expression of Ocn, Opn, and Runx2 in BMSCs transfected with overexpression of GCN5 in the control group or shRNA of GCN5 in the 0.9% group. **(G)** Semi-quantitative and statistical analyses of the results in Panel B. Values are plotted as mean ± SD, *p < 0.05, **p < 0.01.

To further demonstrate that the increased level of GCN5 caused by mechanical strain can promote osteogenic differentiation, we transfected the empty vector, the GCN5 lentiviral overexpression vector, and the knockdown GCN5 vector into the BMSCs (Supplementary Figure S2 and Supplementary Table S2). Elastic mechanical strain was applied to the titanium specimen of BMSCs which were transfected with the empty vector and the knockdown GCN5 vector. BMSCs in the non-strain group were transfected with the GCN5 lentiviral overexpression vector. We used the ALP staining experiment to detect ALP activity. The expression levels of GCN5 and osteogenic differentiation markers OCN, OPN, and RUNX2 were determined by Western blot and real-time PCR. The results of the ALP staining experiments showed that knockdown of GCN5 significantly inhibited the ALP activity of BMSCs exposed to mechanical strain. However, in the control group without mechanical strain, the overexpression of GCN5 saved partial ALP activity (Figure 5A). The results of Western blot and real-time PCR showed that in the 0.9% group, osteogenesis-related markers were significantly repressed with knockdown of GCN5. At the same time, the overexpression of GCN5 could increase the levels of osteogenesis-related markers in the control group. And the overexpression of GCN5 possessed almost the same bone-promoting ability as mechanical strain (Figures 5B–G). These results indicate that GCN5 is a key node for mechanical strain to promote osteogenic differentiation.

### The Influence of GCN5 Regulated by Cyclic Mechanical Stress on Wnt/β-Catenin Signaling Pathway

**FIGURE 6:**
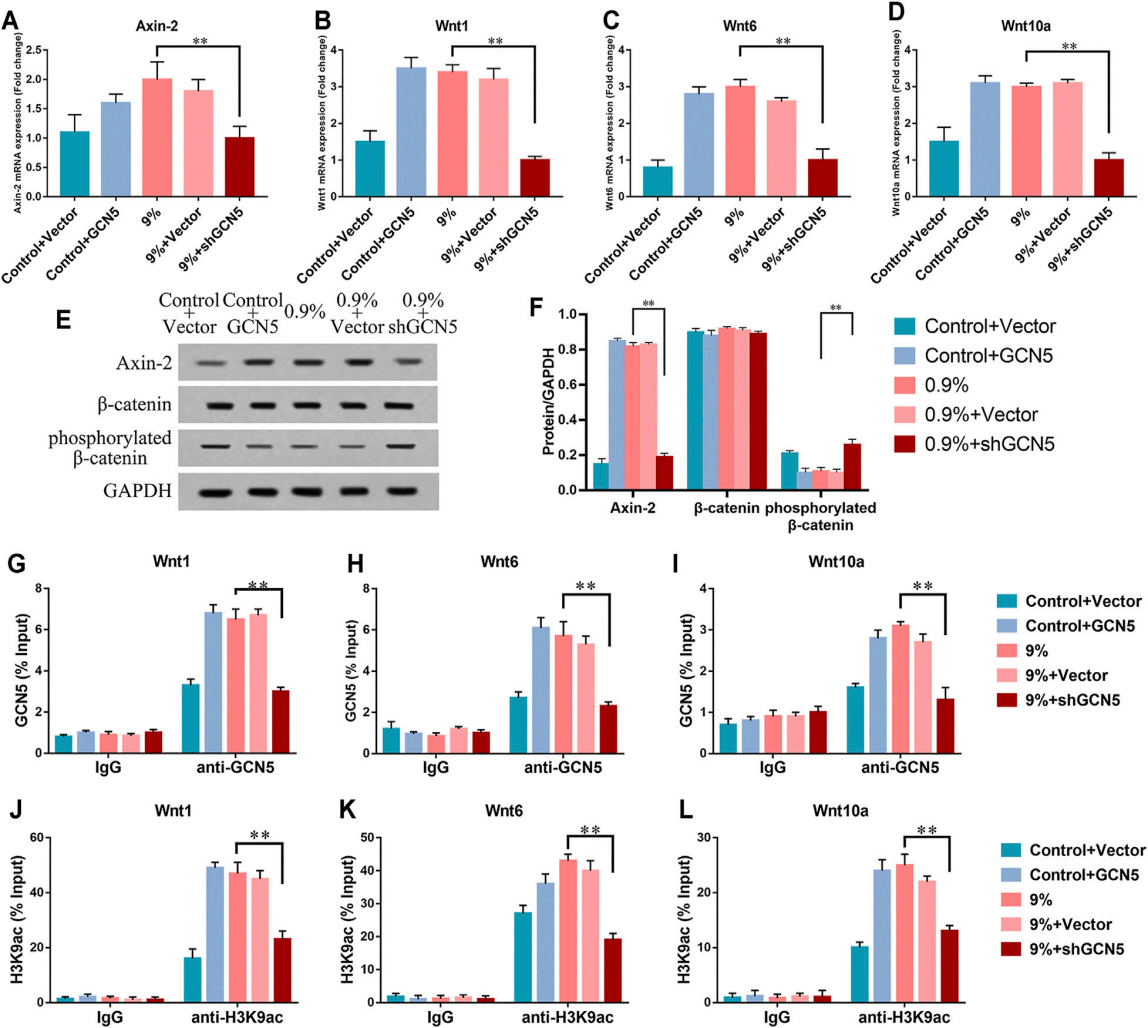
The activity of the Wnt/β-catenin signaling pathway was suppressed by the knockdown of GCN5. **(A–D)** mRNA expression of Axin-2, Wnt1, Wnt6, and Wnt10a in BMSCs transfected with overexpression of GCN5 in the control group or shRNA of GCN5 in the 0.9% group. **(E)** Western blots of Axin-2, phosphorylated β-catenin, and β-catenin in BMSCs transfected with overexpression of GCN5 in the control group or shRNA of GCN5 in the 0.9% group. GAPDH is shown as the loading control. **(F)** Semi-quantitative and statistical analyses of the results in Panel E. **(G–I)** The ChIP assay was performed to evaluate the combination of GCN5 to Wnt1, Wnt6, and Wnt10a promoters after the knockdown of GCN5or overexpression of GCN5. **(J–L)** The ChIP assay was performed to evaluate the enrichment of H3K9ac to Wnt1, Wnt6, and Wnt10a promoter regions. Values are plotted as mean ± SD, *p < 0.05, **p < 0.01.

To further verify that high levels of GCN5 caused by mechanical strain can enhance the Wnt pathway, the levels of markers associated with the Wnt pathway were measured at the same time. In the 0.9% group, the levels of phosphorylated β-catenin were significantly increased and Axin-2 was repressed by the knockdown of GCN5 (Figures 6E,F). The real-time PCR assay showed knockdown of GCN5 inhibited the transcription of related Wnt gene and Axin-2 (Figures 6A–D). In the control group without mechanical strain, the overexpression of GCN5 increased the expression level of Axin-2, repressed the expression of phosphorylated β-catenin (Figures 6E,F), and also promoted the transcription of the Wnt gene and Axin-2 (Figures 6A–D). The ChIP-qPCR directly demonstrated that knockdown of GCN5 could inhibit the enrichment of GCN5 and H3K9ac in the promoter regions of Wnt1, Wnt6, and Wnt10a under the circle mechanical strain (Figures 6G–I).

**FIGURE 7:**
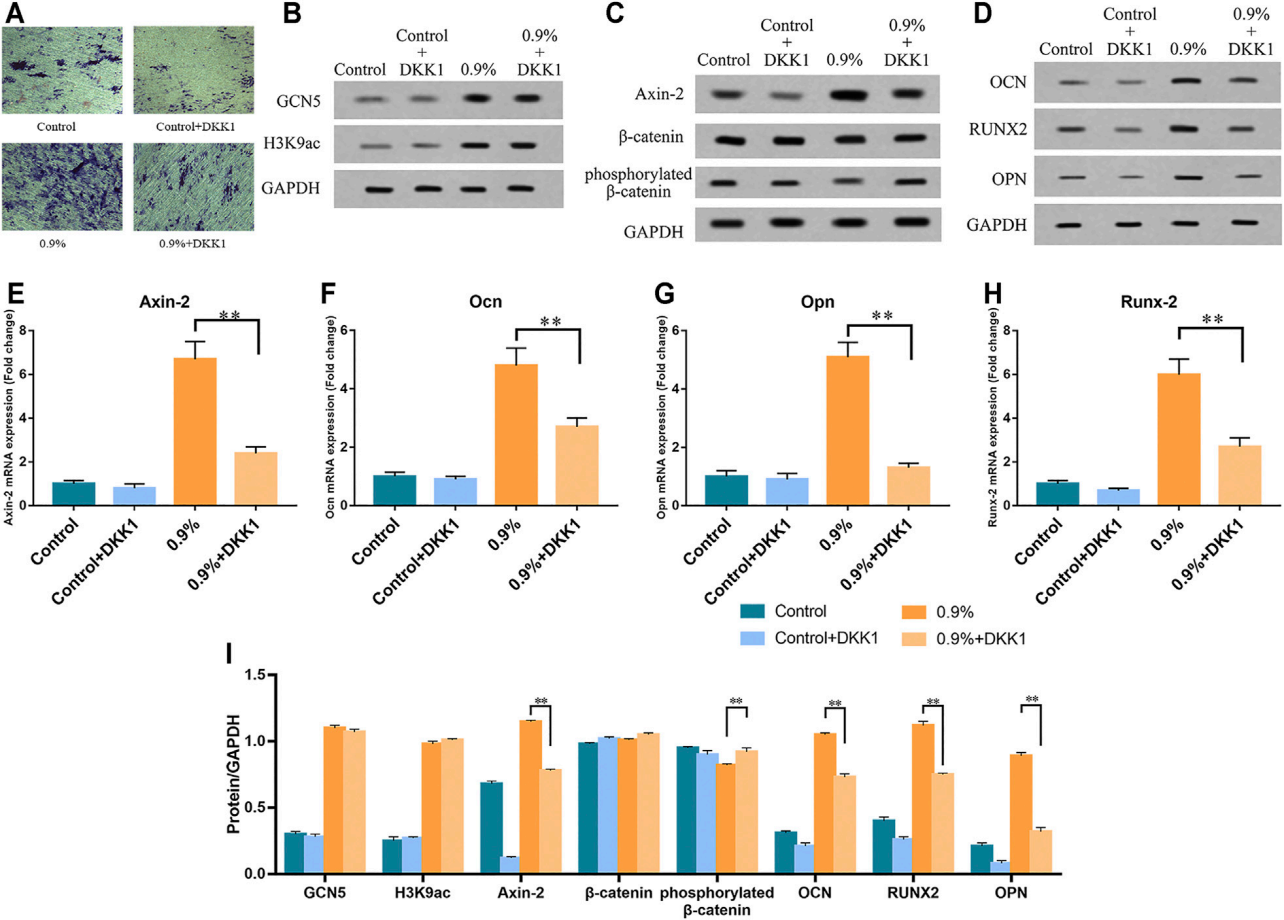
DKK1 inhibited the activity of Wnt/β-catenin signaling pathway and osteogenic differentiation of BMSCs but showed no effect on the expression of GCN5 and H3K9 acetylation modification. **(A)** ALP activity in BMSCs with DKK1 treatment and non–DKK1 treatment. Scale bars: 500 μm. **(B)** Western blots of GCN5 and H3K9ac. **(C)** Western blots of Axin-2, phosphorylated β-catenin, and β-catenin. **(D)** Western blots of Ocn, Opn, and Runx2 in BMSCs with DKK1 treatment and non–DKK1 treatment. **(E–H)** The mRNA expression of Axin-2, Ocn, Opn, and Runx2 in BMSCs with DKK1 treatment and non–DKK1 treatment. **(I)** Semi-quantitative and statistical analyses of the results in Panels **B–D**. Values are plotted as mean ± SD, *p < 0.05, **p < 0.01.

**FIGURE 8:**
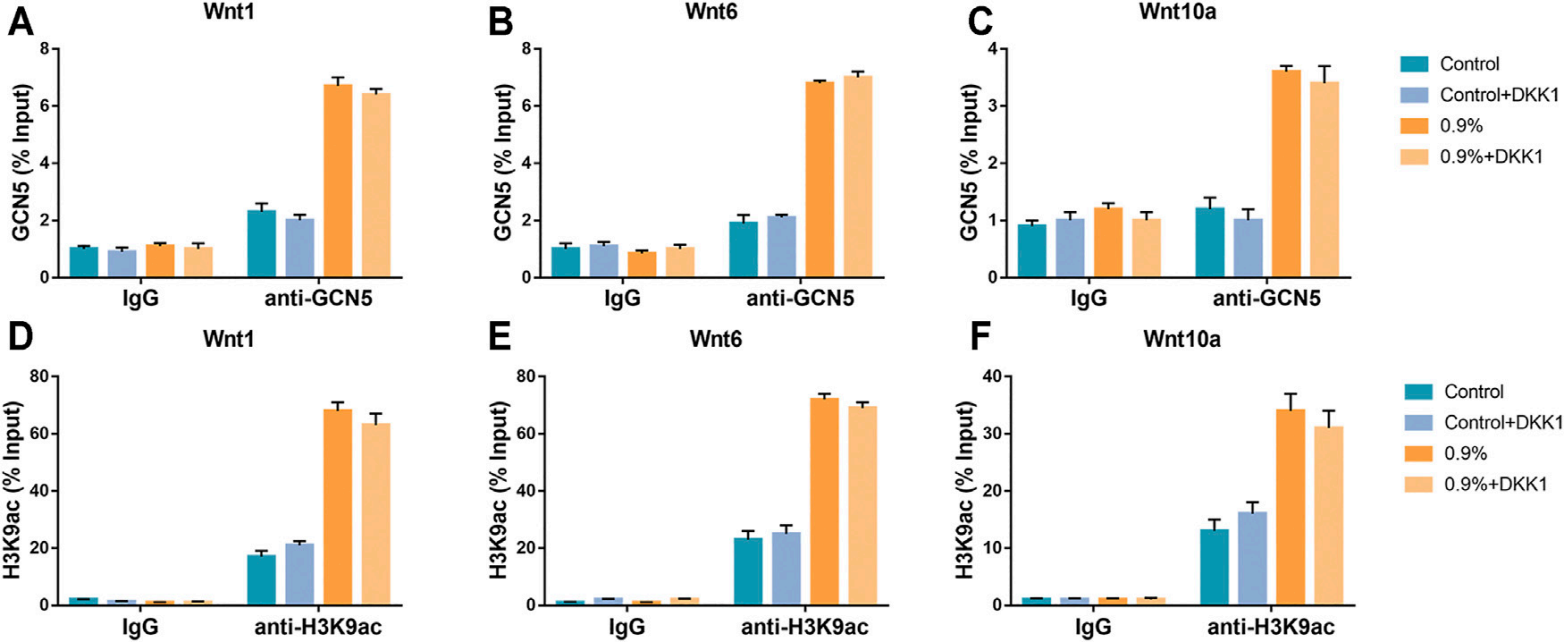
DKK1 could not inhibit GCN5 function. **(A–C)** The ChIP assay was performed to evaluate the combination of GCN5 to Wnt1, Wnt6, and Wnt10a promoters with DKK1 treatment and non–DKK1 treatment. **(D–F)** The ChIP assay was performed to evaluate the enrichment of H3K9ac to Wnt1, Wnt6, and Wnt10a promoter regions with DKK1 treatment and non–DKK1 treatment. Values are plotted as mean ± SD, *p < 0.05, **p < 0.01.

We further verified whether GCN5 which was stimulated by mechanical strain regulated osteogenic differentiation of BMSCs *via* the Wnt pathway. As an inhibitor of the Wnt pathway, DKK1 binds to membrane receptors and prevents Wnt ligands from binding to membrane surface receptor complexes, thereby inhibiting Wnt signaling pathway activity and osteogenic differentiation of BMSCs (Scott et al., 2013). We assessed ALP activity on BMSCs with DKK1 treatment and non–DKK1 treatment. As a result, DKK1 significantly suppressed ALP activity in the control group as well as in the 0.9% group (Figure 7A). The Western blot and real-time PCR results showed that DKK1 reduced the activity of the Wnt signaling pathway during mechanical strain application. The expression of Axin-2 in the pathway decreased and phosphorylated β-catenin increased due to DKK1 (Figures 7C,E). Not only the activity of the Wnt signaling pathway but also the expression of downstream osteogenic differentiation markers was repressed, including Opn, Ocn, and Runx2 (Figure 7D). However, DKK1 did not significantly affect the levels of GCN5 and H3K9ac in terms of protein expression (Figure 7B). The results of the ChIP analysis demonstrated that DKK1 had no significant effect on the enrichment of GCN5 and H3K9ac in the promoter regions of Wnt1, Wnt6, and Wnt10a. After mechanical strain stimulation, the enrichment of GCN5 and H3K9ac in the Wnt1, Wnt6, and Wnt10a promoter regions was still increased compared to the control group (Figure 8). These results indicate that DKK1 can only affect the downstream Wnt/β-catenin pathway activity but not upstream GCN5. Combined with the abovementioned results of overexpression and knockdown of GCN5, it is indicated that the high level of GCN5 caused by mechanical strain regulates the osteogenic differentiation of BMSCs through the Wnt/β-catenin signaling pathway.

## Discussion

**FIGURE 9:**
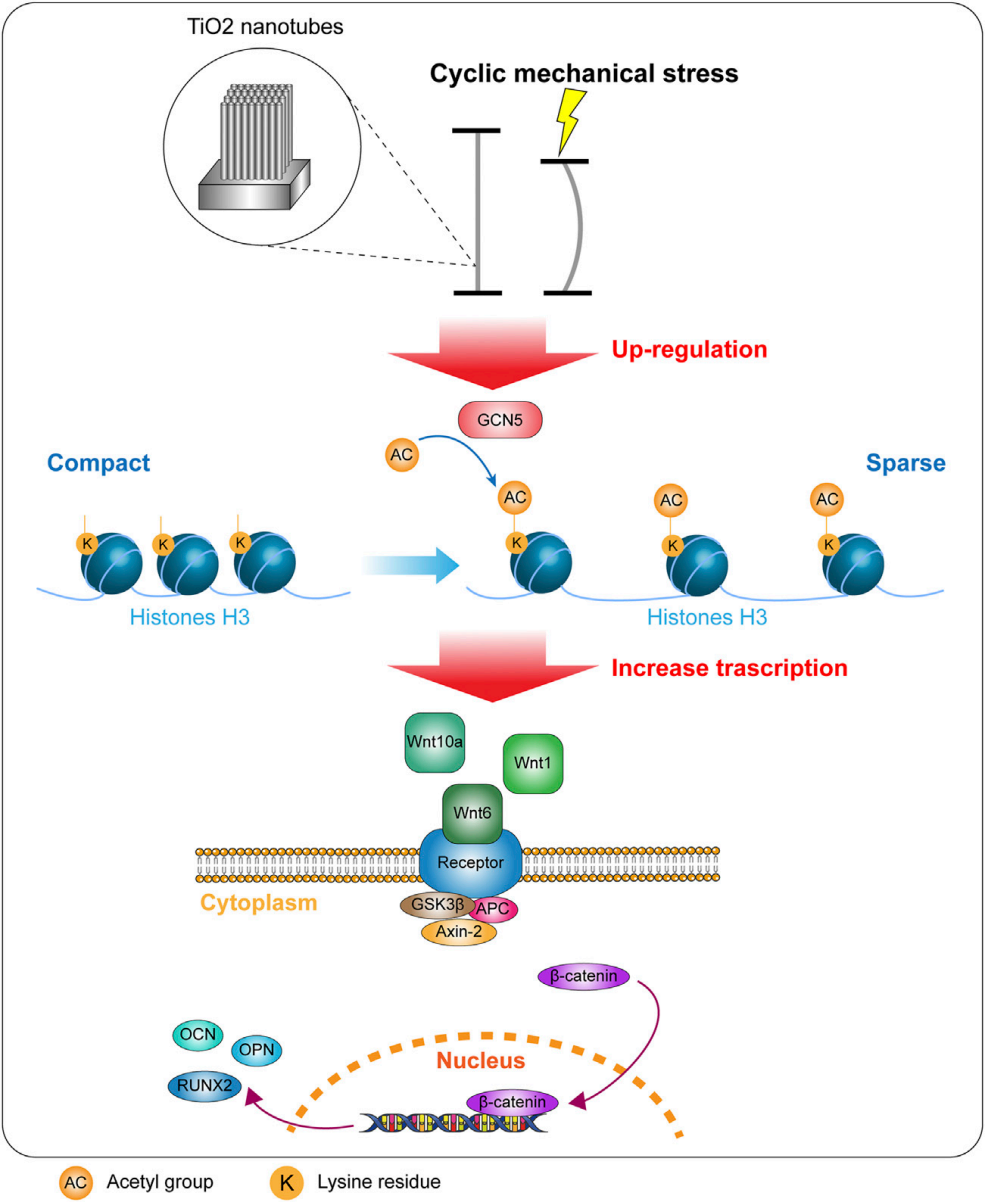
Cyclic mechanical strain upregulated the expression of GCN5 and promoted the level of acetylation of H3K9, thereby increased transcription of related Wnt ligands and activating the Wnt signaling pathway.

Titanium and titanium alloys are the most widely used metal materials in orthopedic clinical implants due to the good properties of titanium (Szuhanek et al., 2020). In the application of internal implants, whether BMSCs on the surface of titanium prosthesis can differentiate into osteoblasts is one of the most critical issues determining the osseointegration effect (Wu et al., 2020). Previous studies have shown that the modification or fixation of surface coatings and biological functional molecules will facilitate osseointegration. Recently, due to the study of cell response to physical signals, the mechanical stress on the implant has attracted the attention of many researchers (Lee et al., 2020). BMSCs have a variety of differentiation potentials, and many researchers have indicated that differentiation and phenotypic expression of BMSCs can be affected by mechanical signals (Wang et al., 2016; Huang et al., 2017). Titanium can undergo extremely fine deformation, and the mechanical strain signal that caused this deformation can affect the signal pathway activity of surface BMSCs and change the direction of differentiation (de Peppo et al., 2014). However, the mechanism is very complicated and involves multiple signal paths, which is one of the current research focuses. In order to achieve a better titanium specimen deformation effect, we have designed a cyclic mechanical strain loading device. In our previous study, we confirmed the relevant parameters of the surface morphology of TiO_2_-modified titanium metal specimen. Moreover, we have demonstrated that cyclic mechanical strain that causes deformation of titanium specimen can promote osteogenic differentiation of BMSCs on the surface of TiO_2_ nanotube–modified titanium. We set up experiments on the time, frequency, and amplitude of cyclic mechanical strain in the early stage and obtained the optimal parameters for promoting osteogenic differentiation of BMSCs in this device, and applied it to this study. 0.3 and 0.9% represent the degree of compression of the specimen after being subjected to cyclic mechanical stress. As the amplitude of cyclic mechanical stress increases, the osteogenic differentiation of surface cells gradually improved (Chang et al., 2019). In this study, we demonstrated the ability of cyclic mechanical regulated stress osteogenic differentiation *via* the GCN5/Wnt/β-catenin signaling pathway (Figure 9).

Epigenetics refers to the activation or inhibition of a gene without altering the DNA sequence (Richards, 2009). Histone acetylation modification is an important mode of regulation in epigenetics. Histone acetylation acts as a signal that alters the interaction between histones and the adjacent nucleosomes and between histones and transcription factors, thereby affecting the structure of the nucleosomes, producing a more open or closed chromatin environment that affects the transcription process of genes. The activation and inhibition of the signaling pathways are closely related to this change. Histone acetylation modification is regulated by the equilibrium relationship between HATs and HDACs. HDACs can reverse the acetylation of the N-tail lysine residue of the core histone, resulting in chromatin condensation and inhibition of gene transcription. On the contrary, HAT transfers the acetyl group of acetyl-CoA to the specific lysine residue at the amino terminus of histones, loosening the chromatin structure and promoting transcription of the gene (Han et al., 2016). Previous literature has reported that HAT played an important role in bone formation and bone loss (Szuhanek et al., 2020; Wu et al., 2020). H3K9 is prone to be acetylated and is a key site for acetylation modification (Zhang et al., 2016). H3K9 becomes H3K9ac after being acetylated, which affects the activation of the promoter region of related genes. Therefore, elevated levels of histone acetylation can promote gene transcription that regulates osteogenic differentiation of BMSCs, such as the Wnt signaling pathway. Previous studies have focused on the role of HDACs in cellular signaling pathways, but there are few studies on the role of the HATs family in regulating the cellular signaling pathways. In terms of mechanical signals, there are no reports on the regulation of the cellular signaling pathways by the HATs family.

GCN5 is named after the conventional signaling pathways that regulate amino acid synthesis originally found in yeast (Weake, 2020). Yeast strains with GCN5 mutations when lacking amino acids cannot de-inhibit a large number of amino acid biosynthesis genes. Subsequently, studies have confirmed that GCN5 is a transcription-related HAT. From yeast to humans, GCN5 shows highly evolutionarily conserved enzyme specificity. It has been reported in the literature that GCN5, as an important member of the HATs family, plays an important role in regulating cellular signaling pathways (Zhao et al., 2018). In U87 and U251 cell lines, reducing GCN5 expression by siRNA interference can downregulate the expression of AKT/ERK and upregulate the expression of p21, thereby inhibiting cell proliferation, invasion, and colony formation. In the dental pulp inflammation microenvironment, GCN5 can partially restore the inhibitory effect of inflammation on dental pulp stem cells by activating the Wnt/β-catenin signaling pathway (Arede et al., 2020). In this study, we proved that the increase in cyclic mechanical stress caused an upregulation of the expression level of GCN5. This indicates that GCN5 in BMSCs can sense and change in response to mechanical signal stimulation outside the cells.

The Wnt gene family encodes secreted glycoproteins, which act as ligands for the Frizzled family of transmembrane receptor proteins. In many types of cells, the Wnt/β-catenin signaling pathway plays a role in cell proliferation, cell-fate determination, and cell differentiation. Wnt ligand binding to Frizzled receptor leads to the downregulation of GSK3β. GSK3β can phosphorylate β-catenin and then β-catenin could be hydrolyzed by APC complex. Its downregulation makes β-catenin accumulate in the cytoplasm, which then migrates into the nucleus and when combined with TCF/LEF transcription factors affects the transcription of target genes (Weglage et al., 2020). The Wnt/β-catenin signaling pathway is an important pathway in the process of cell differentiation. In the neural stem cell model of Alzheimer’s disease, specifically activating the Wnt/β-catenin signaling pathway can promote the proliferation of neural stem cells, differentiate them into more neurons, and reduce neuronal apoptosis (Demirci et al., 2020). The canonical Wnt pathway is especially influential in inhibiting the expression of the major adipogenic inducers, PPARγ and CCAAT/enhancer-binding protein α, to suppress adipogenic differentiation while upregulating the osteogenic regulators Runx2, Dlx5, and Osterix (Kang et al., 2007). In this study, we found that GCN5 activates acetylation of H3K9 and the Wnt signaling pathway when BMSCs were exposed to mechanical strain. As the degree of deformation of TiO_2_ nanotube–modified titanium specimen aggravates, the expression level of GCN5 gradually increases. Due to the acetylation modification of GCN5, the transcription levels of wnt1, wnt6, and wnt10a and the expression levels of Axin-2 and β-catenin also increased. DKK1 is a specific inhibitor of the Wnt pathway and can inhibit Wnt signaling by competing with the ligand Wnt for receptor LRP6 (Min Swe et al., 2020). DKK1 could still inhibit the level of Wnt signaling pathway under mechanical strain, but it did not inhibit GCN5 and its target H3K9ac. Cyclic mechanical stress can still increase the expression level of GCN5 and the level of acetylation modification. Therefore, we further proved the regulatory relationship between GCN5 and the Wnt/β-catenin signaling pathway through the intervention of DKK1. This may provide a research basis for the development of novel drugs to improve the combination of implants and host bones in the future.

In conclusion, it was revealed that the cyclic mechanical strain enhanced osseointegration on TiO_2_ nanotube–modified titanium specimen surface through GCN5/Wnt. Cyclic mechanical strain promoted the expression of GCN5 and increased the level of acetylation of H3K9, thereby activating the Wnt signaling pathway. This study suggests that applying cyclic mechanical stress to the prosthetic implants could be more effective in promoting osseointegration and improving patient prognosis, and it is a promising prospect for the clinical treatment of bone defects and arthroplasty. This also provides a relevant basis and reference for postoperative rehabilitation training.

## Data Availability

The original contributions presented in the study are included in the article/Supplementary Material, and further inquiries can be directed to the corresponding authors.
