# Supplementary material for: Cyclic Mechanical Strain Regulates Osteoblastic Differentiation of Mesenchymal Stem Cells on TiO2 Nanotubes Through GCN5 and Wnt/β-Catenin
Source: Front Bioeng Biotechnol. 2021 Nov 15;9:735949. doi: 10.3389/fbioe.2021.735949 (PMC8634263; doi:10.3389/fbioe.2021.735949)
Supplement: Supplementary file 1 [file Table1.docx]

Supplementary Table 1

The selected primers for Wnt1, Wnt6 and Wnt10a promoter sequences

| Genes | Forward(5'-3') | Reverse(5'-3') | Availability |
| --- | --- | --- | --- |
| Wnt1 | AACTCCACCCATGCTCTGT | GCTGTGGTCCCTTCTCTTCC | sclected |
|  | CCAGTAGGGCATGCAAACTG | AAGCTGACATGCAAGTCCAA | unselected |
|  | TAAGCAGAACCTGACTGA | AGCCTAGACAAGTCAGTA | unselected |
| Wnt6 | CAGGGACCCGTAGACAAGTG | TATTGGGGGCGGACAGTGTA | sclected |
|  | TACGAAAGCTAGAAGCTA | GGAATCGACCTAGCTAGC | unselected |
|  | CGTTAGCTCCTAGCTAGCTA | TTAGCTTCAGAATCGACCTC | unselected |
| Wnt10a | CATTCAGGTTAGGGCCCCAG | CACATTTGTCTTTGGGCTTCATCT | sclected |
|  | GAATAGTCGCCTAGAATCG | TTACGATCCATTGATCTAGC | unselected |
|  | ACGCATCGAATCCTAGCAT | GCGCTAGCTAGCCCTAGCTA | unselected |
