# Supplementary figures and images for: Cyclic Mechanical Strain Regulates Osteoblastic Differentiation of Mesenchymal Stem Cells on TiO2 Nanotubes Through GCN5 and Wnt/β-Catenin
Source: Front Bioeng Biotechnol. 2021 Nov 15;9:735949. doi: 10.3389/fbioe.2021.735949 (PMC8634263; doi:10.3389/fbioe.2021.735949)

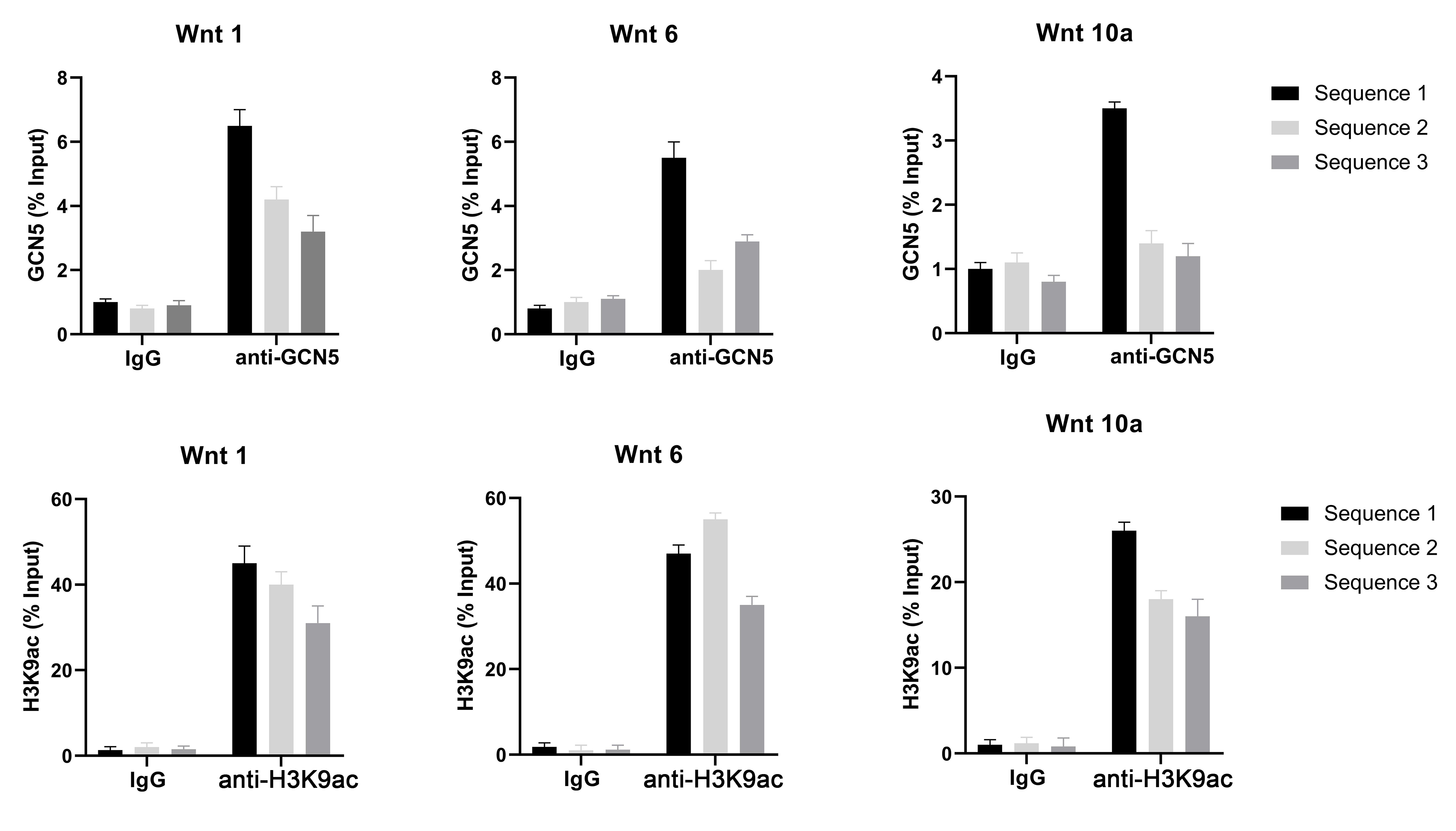

Supplement: Supplementary file 2 [file Image1.JPEG]

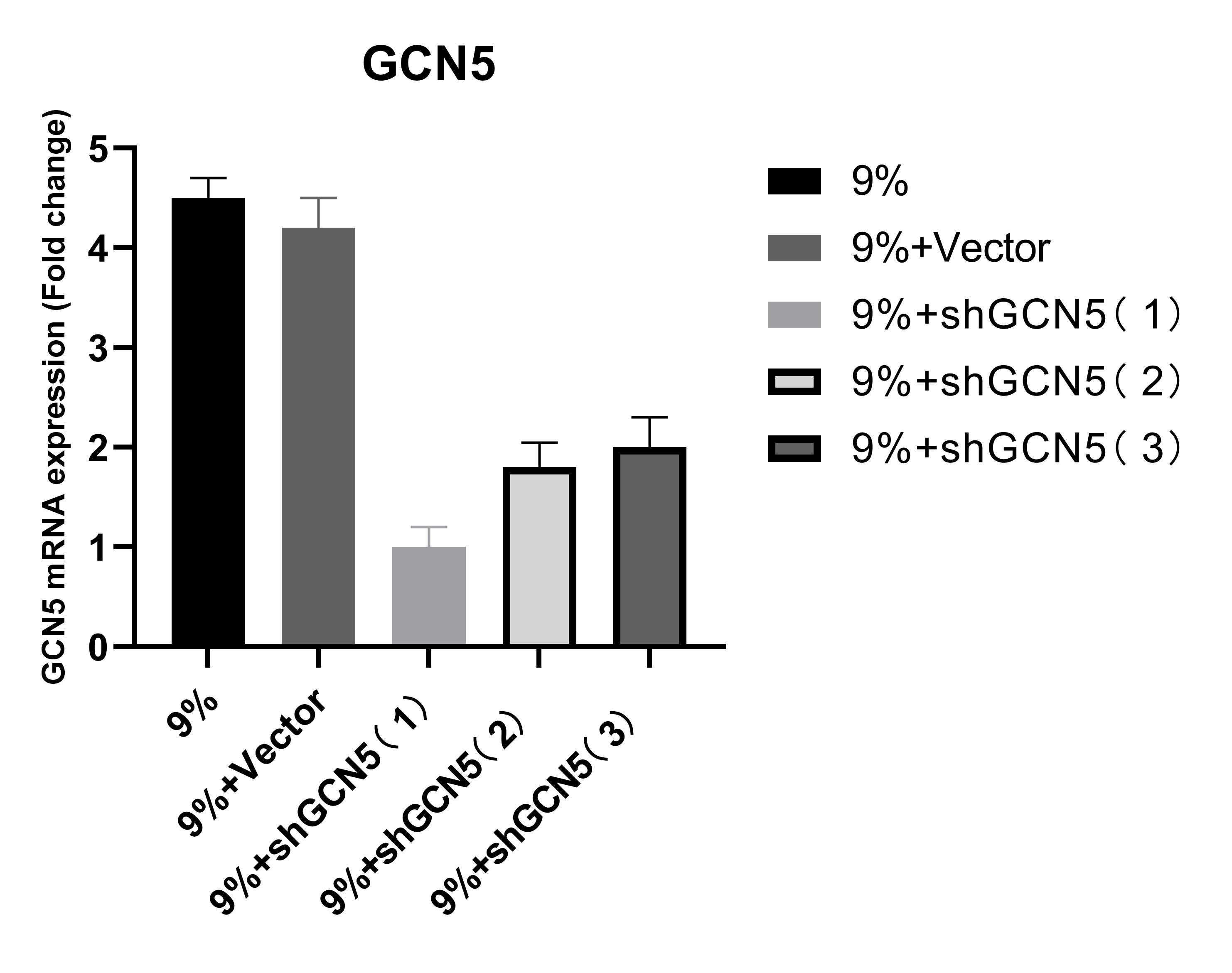

Supplement: Supplementary file 3 [file Image2.JPEG]
