# Supplementary material for: Cyclic Mechanical Strain Regulates Osteoblastic Differentiation of Mesenchymal Stem Cells on TiO2 Nanotubes Through GCN5 and Wnt/β-Catenin
Source: Front Bioeng Biotechnol. 2021 Nov 15;9:735949. doi: 10.3389/fbioe.2021.735949 (PMC8634263; doi:10.3389/fbioe.2021.735949)
Supplement: Supplementary file 4 [file Table2.docx]

Supplementary Table 2

The selected primers for GCN5 shRNA

| Genes | Forward(5'-3') | Reverse(5'-3') | Availability |
| --- | --- | --- | --- |
| shRNA | CCGGGCAGGGTGTTCTGAACTTT | ATTCAAAAAAGCAGGGTGTTCT | sclected |
| （1） | CTCAAGAGAAAAGTTCAGAACA | GAACTTTTCTCTTGAGAAAGTTC |  |
| shRNA | GCTAATCCCGATCGCAAATCGC | AATTCAAAAAGACCACGAATGC | unselected |
| （2） | AGCCCTAGATATTTAGCTAGTTA | CCAAGGAATTCTCTTGAAATTCC |  |
| shRNA | CGGGGCCGATATCTGGGCTACA | AATTCAAAGCCGCCGATATCTG | unselected |
| （3） | TTCAAGAGAATGTAGCCCAGAT | GCTACATTCTCTTGAAATGTAGC |  |
